# Milk as an Article of Diet

**Published:** 1881-12

**Authors:** 


### MILK AS AN ARTICLE OF DIET.

The digestibility of milk depends, in many instances, upon the temperature at which it is taken into the stomach. Few seem rightly to appreciate the value of heat introduced into the system as a vital restorative. No more acceptable mode of accomplishing this can be devised than the drinking of hot milk. Milk heated to much above 100° Fahr. loses for a time a degree of its sweetness and its density. I am persuaded, however, that no one who, fatigued by over-exertion of body or mind, has ever experienced the reviving influence of this beverage, heated as hot as it can be sipped, will willingly forego a resort to it because of its having been rendered somewhat less acceptable to the palate. The promptness with which its cordial influence is felt is indeed surprising. Some portion of it seems to be digested and appropriated almost immediately; and I am certain that many who now fancy they need alcoholic stimulants when exhausted by fatigue, will find in this simple draught an equivalent that shall be abundantly satisfying, and far more enduring in its effects.—*Med. Record,* p., 315, 1881.

